# Hormonal Responses to Cholinergic Input Are Different in Humans with and without Type 2 Diabetes Mellitus

**DOI:** 10.1371/journal.pone.0156852

**Published:** 2016-06-15

**Authors:** Sara Chowdhury, Songyan Wang, Judit Dunai, Rachel Kilpatrick, Lauren Z. Oestricker, Michael J. Wallendorf, Bruce W. Patterson, Dominic N. Reeds, Burton M. Wice

**Affiliations:** 1 Department of Internal Medicine, Division of Endocrinology, Metabolism and Lipid Research Washington University School of Medicine, Saint Louis, MO, United States of America; 2 Division of Biostatistics, Washington University School of Medicine, Saint Louis, MO, United States of America; 3 Department of Internal Medicine, Division of Nutritional Science, Metabolism and Lipid Research Washington University School of Medicine, Saint Louis, MO, United States of America; Harvard Medical School, UNITED STATES

## Abstract

Peripheral muscarinic acetylcholine receptors regulate insulin and glucagon release in rodents but their importance for similar roles in humans is unclear. Bethanechol, an acetylcholine analogue that does not cross the blood-brain barrier, was used to examine the role of peripheral muscarinic signaling on glucose homeostasis in humans with normal glucose tolerance (NGT; n = 10), impaired glucose tolerance (IGT; n = 11), and type 2 diabetes mellitus (T2DM; n = 9). Subjects received four liquid meal tolerance tests, each with a different dose of oral bethanechol (0, 50, 100, or 150 mg) given 60 min before a meal containing acetaminophen. Plasma pancreatic polypeptide (PP), glucose-dependent insulinotropic polypeptide (GIP), glucagon-like peptide-1 (GLP-1), glucose, glucagon, C-peptide, and acetaminophen concentrations were measured. Insulin secretion rates (ISRs) were calculated from C-peptide levels. Acetaminophen and PP concentrations were surrogate markers for gastric emptying and cholinergic input to islets. The 150 mg dose of bethanechol increased the PP response 2-fold only in the IGT group, amplified GLP-1 release in the IGT and T2DM groups, and augmented the GIP response only in the NGT group. However, bethanechol did not alter ISRs or plasma glucose, glucagon, or acetaminophen concentrations in any group. Prior studies showed infusion of xenin-25, an intestinal peptide, delays gastric emptying and reduces GLP-1 release but not ISRs when normalized to plasma glucose levels. Analysis of archived plasma samples from this study showed xenin-25 amplified postprandial PP responses ~4-fold in subjects with NGT, IGT, and T2DM. Thus, increasing postprandial cholinergic input to islets augments insulin secretion in mice but not humans.

***Trial Registration*:** ClinicalTrials.gov NCT01434901

## Introduction

Transmitters and peptides released from neurons that innervate islets play important roles in regulating insulin and glucagon release [1,2]. In general, parasympathetic and sympathetic neurons that innervate pancreatic islets increase and inhibit insulin release, respectively [1–4]. In rodents, islets are richly innervated by parasympathetic neurons [5]. Previous studies from our laboratory have shown that a cholinergic neural relay amplifies the effects of glucose-dependent insulinotropic polypeptide (GIP) on insulin release in mice [6]. Studies by others using genetically modified mice and/or islets indicate that cholinergic signaling via M3 muscarinic acetylcholine receptors plays an important role in regulating insulin and glucagon release [7–12]. Consistent with mouse experiments, studies with the isolated perfused human pancreas have shown that electrical stimulation of the splanchnic nerve in the presence and absence of selective neural inhibitors increases both cholinergic and sympathetic input to islets which in turn, regulates insulin, glucagon, pancreatic polypeptide (PP), and somatostatin release [13–18]. Further, neurotransmitters regulate insulin release in isolated human islets [19]. In contrast to the in situ and ex vivo studies, physiologic stimuli (e.g. nutrients, stress) would differentially affect parasympathetic versus sympathetic input to islets. Thus, the physiologic relevance of the electrical stimulation and human islet studies is not clear.

There are conflicting reports on the effects of physiologic levels of cholinergic signaling for regulating insulin and glucagon responses in vivo in humans. For example, prior prolonged mild hyperglycemia results in a compensatory increase in C-peptide secretion during intravenous glucose tolerance tests, which is only partially inhibited by atropine [20]. In another study, atropine inhibited the cephalic insulin response to meal ingestion by 20% [21] Specific anti-psychotic medications that are associated with development of T2DM also exhibit secondary affinity/antagonism to muscarinic M3 receptors [22]. During 50-gram oral glucose tolerance tests, areas under the curve for glucose, glucagon-like peptide-1 (GLP-1), and insulin secretion rates (ISRs) were increased in humans with truncal vagotomy plus pyloroplasty compared to controls [23]. However, these changes are likely indirect because vagotomy also increased the rate of gastric emptying. Conversely, vagotomy for peptide ulcer disease had little effect on plasma glucose levels following intravenous administration of glucose [24,25] and atropine inhibited postprandial PP release but not insulin secretion in Pima Indians [26]. Thus, the importance of cholinergic regulation of insulin and glucagon release in response to a physiologic mixed meal in humans is unclear.

A recent study suggested that in contrast to mice, human islets are poorly innervated by parasympathetic (cholinergic) neurons [5]. If so, a neural cholinergic relay to islets would have little effect on islet physiology. PP is a 36-amino acid peptide produced by a subpopulation of endocrine cells called PP cells. Circulating PP is undetectable in humans after total pancreatectomy indicating it is produced almost exclusively by the pancreas [27]. Although there are species-specific differences [28], in humans PP cells are mainly localized at the periphery of islets [29–31]. PP is released into the circulation in response to meal ingestion [32] but not to intravenous infusion of glucose, amino acids, or fat [27,33]. Atropine blocks PP release in response to food intake, insulin-induced hypoglycemia, and intravenous infusion of GIP, bombesin, gastrin releasing peptide, neurotensin, and bethanechol [34–38]. Truncal vagotomy abolishes PP release in most cases studied [34,39,40] but a non-vagal mechanism may also contribute to the regulation of PP release [41]. These collective results suggest that PP secretion is regulated by vagal and non-vagal cholinergic input to islets.

Xenin-25 is an intestinal peptide reportedly produced by a subset of enteroendocrine cells [42–45]. Effects of xenin-25 are mediated by activation of neurotensin receptor 1 [46–51]. We have shown that in sections of human pancreas, neurotensin receptor 1 is detectable on nerves, but not islet endocrine cells [33]. Further, during graded glucose infusions, administration of xenin-25, alone and more so when co-administered with GIP, profoundly increased PP release in humans [33]. These results strongly suggest that functional cholinergic neurons innervate human as well as rodent islets. Bethanechol is a charged quaternary amine moiety that does not cross the blood brain barrier and can thus be used to study the effects of muscarinic cholinergic signaling in the periphery. The purpose of the present study is to determine if bethanechol and/or xenin-25 increase postprandial cholinergic input to islets and if this signaling is associated with changes in postprandial glucose, GIP, GLP-1, insulin, C-peptide, glucagon, and PP levels and insulin secretion rates (ISRs) in humans with normal glucose tolerance (NGT), impaired glucose tolerance (IGT) and T2DM. Results indicate that increasing postprandial cholinergic signaling to islets by administration of bethanechol or xenin-25 has little net effect on insulin or glucagon release.

## Materials and Methods

### Bethanechol Study

#### Human Subjects

The study protocol was approved by Washington University’s Human Research Protection Office and the FDA (IND#103,374) and was registered with ClinicalTrials.gov (NCT01434901). Studies were performed by the nursing and medical staff in the Clinical Research Unit of the Institute of Clinical and Translational Sciences of Washington University after obtaining written informed consent. Subject recruitment was initiated on August 15, 2011 and follow-up for the final participant was completed on July 7, 2014. Subjects were recruited through Washington University’s Research Participant Registry as well as from the PI’s database of previous participants. After a phone screen, potential participants underwent a screening visit in the Clinical Research Unit. Subjects were remunerated to encourage completion of the study. Male and female subjects with NGT (n = 10), IGT (n = 11), and mild T2DM (n = 9) were studied. Glucose tolerance was defined by the 2-hour plasma glucose level measured during a 75-gram oral glucose tolerance test (OGTT) using diagnostic criteria of the American Diabetes Association [52]. With respect to T2DM, selection criteria were designed to exclude subjects with advanced beta cell failure: subjects were required to have HbA1c ≤ 9%, could not be using insulin for treatment, had no known history of symptomatic gastroparesis or peripheral neuropathy (e.g., burning or tingling in feet) and were enrolled only if oral anti-diabetic medications could be safely discontinued for 48-hours before each study visit. Women of childbearing potential were required to use birth control. Subjects were excluded if they 1) had a history of chronic pancreatitis and/or risk factors for chronic pancreatitis 2) had a history of significant gastrointestinal disorders, (e.g. peptide-ulcer disease) 3) were taking non-diabetes medications known to affect glucose homeostasis and 4) had any significant chronic illness including heart, renal, liver, inflammatory or malignant disease. The use of choline esters is contraindicated in persons with hyperthyroidism, coronary artery disease, peptic ulcer, asthma, chronic bronchitis, or COPD. Subjects with any of these conditions were excluded. Baseline characteristics for each group were determined during a screening visit following a 12-hour fast and are shown in Table 1. Based on variance and correlation estimates from the current study, 10 subjects would provide 80% power when the true differences are 4500 for PP AUC (sd = 5000, correlation = 0.6) and 29,000 for ISR AUC (sd = 31,623, correlation = 0.6).

**Table 1:** Baseline Demographic and Clinical Characteristics for Bethanechol and Xenin-25 Studies.

***Bethanechol Study- NCT01434901***	***NGT (n = 10)***	***IGT (n = 11)***	***T2DM (n = 9)***	***P Val***
*2-hour Glucose (mg/dL)*	128 ±12	165 ± 19	251 ± 31	<0.0001
*Fasting Glucose (mg/dL)*	91 ± 5	95 ± 9	115 ± 20	<0.001
*HbA1c (%)*	5.6 ± 0.2	5.7 ± 0.4	6.2 ± 0.7	<0.02
*HbA1c (mmol/mol)*	37 ± 2.6	39 ± 4.7	44 ± 7.8	<0.02
*BMI (kg/m*^*2*^*)*	32 ± 7	31 ± 6	35 ± 6	NS
*Age (years)*	48 ± 12	45 ± 5	55 ± 9	0.07
*Gender (Males/Females)*	5/5	8/3	3/6	NS
*Fasting PP (pg/mL)*	23 ± 15	23 ± 12	20 ± 13	NS
***Xenin-25 Study- NCT00949663***	***NGT (n = 10)***	***IGT (n = 14)***	***T2DM (n = 12)***	***P Val***
*2-hour Glucose (mg/dL)*	118 ±12	162 ± 14	245 ± 23	<0.0001
*Fasting Glucose (mg/dL)*	95 ± 7	97 ± 7	127 ± 20	<0.0001
*HbA1c (%)*	5.6 ± 0.3	5.7 ± 0.3	6.2 ± 0.6	<0.001
*HbA1c (mmol/mol)*	38 ± 2.2	39 ± 1.9	44 ± 4.3	<0.001
*BMI (kg/m*^*2*^*)*	29 ± 5.1	31 ± 5.3	38 ± 5.7	<0.01
*Age (years)*	40 ± 11	46 ± 9.1	51 ± 6.4	<0.05
*Gender (Males/Females)*	4/6	9/5	6/6	NS
*Fasting PP (pg/mL)*	28 ± 17	24 ± 18	19 ± 10	NS

Group values ± SD are shown for the bethanechol (top) and xenin-25 (bottom) studies. P values were determined by one-way ANOVA for continuous variables and by Fisher exact test for categorical variables. Note that in the xenin-25 study, PP levels were measured in only 10 subjects per group.

#### Study Design

**Fig 1:**
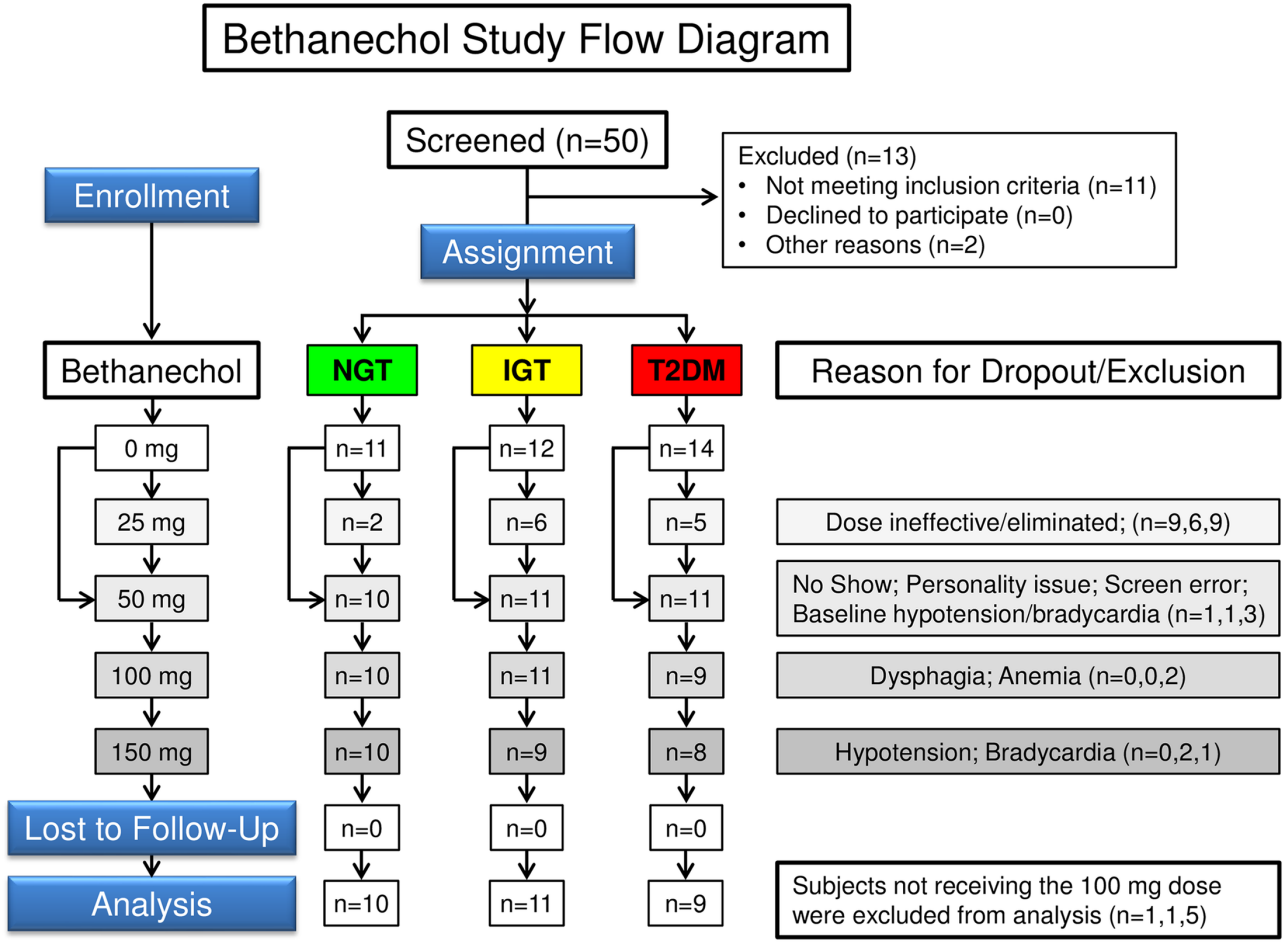
Flow diagram for bethanechol study. Diagram is for a non-randomized crossover study with escalating doses of bethanechol in humans with NGT, IGT, and T2DM.

This is a crossover study in which each participant underwent a series of meal tolerance tests after a 12-hour overnight fast (Fig 1). Subjects were blinded to treatment. Each visit was separated by at least 2 weeks. Hemoglobin levels were measured before each study visit and anyone with a value <11.2 g/dL had that study delayed. In subjects with T2DM and taking oral diabetes medications, drugs were discontinued for 48 hours before each study visit. An intravenous catheter was placed into a hand vein. This hand was kept warm in a thermostatically controlled box for sampling arterialized venous blood [53,54]. Subjects with a fasting blood sugar ≥ 120 mg/dL were given a bolus of intravenous human insulin (0.01 U/kg) at 30 min intervals as needed to decrease the blood glucose level to 100–120 mg/dL to limit variability of initial glucose levels. Blood glucose concentrations were stable for longer than 20 min before administration of bethanechol.

#### Bethanechol Dosing

Bethanechol is an acetylcholine analog that does not cross the blood-brain barrier. Oral dosing was based on a survey of the literature as follows. Measuring the stretch response of the bladder muscle [55,56], the authors reported a progressive increase in mean cystometric pressure with doses of 50, 100, and 200 mg. Of the 20 subjects in this study, sweating, diarrhea, chills, bradycardia and hypotension were noted in only 1–2 patients with the 200 mg dose. The maximal response to oral bethanechol began after ~1 hour and the action of moderate doses (50–100 mg) persisted for 2–3 hours. Bethanechol has also been used to treat humans with xerostomia after radiation therapy (25–50 mg 3 times per day [57,58]) and to relieve side effects due to tricyclic antidepressants (25 mg 3 times per day [59]). To ensure patient safety in our study, bethanechol was administered in escalating doses of 0, 25, 50, and 100 mg during successive meal tolerance tests. Interim results indicated that the 25 mg dose was ineffective and the 100 mg dose was well-tolerated. Thus, the 25 mg dose was eliminated and the maximal dose was increased to 150 mg. To monitor for symptoms of bethanechol toxicity, subjects were placed on continuous cardiac monitoring with blood pressure and heart rate checked every 15 min. There were no adverse events or unintended effects during this study.

#### Meal Tolerance Tests

Boost Plus (Nestle Health Science, Florham Park, New Jersey) is a liquid mixed meal (360 calories, 14 g of fat, 45 g of carbohydrates, and 14 g of protein). Fasted subjects ingested Boost Plus and liquid acetaminophen (ACM; 1.5 g/15 mL; Q-PAP Infants’ Drop; Qualitest Pharmaceuticals, Huntsville, AL) over a 3 min period starting at 0 min. The ACM was included to allow for estimation of the rate of gastric emptying as discussed in our earlier study [60]. Bethanechol (or placebo) was administered orally with 100 mL of water 1 hour before meal ingestion.

#### Measurements

Complete metabolic profiles and plasma concentrations of glucose, insulin, C-peptide, glucagon, PP, ACM, total GIP, active GLP-1, and HbA1c were determined as previously described [33,60,61]. The PP assay involves an extraction step to remove interfering compounds [33]. A cholinergic symptom survey was administered before, during, and immediately after each study visit. Any diarrheal episodes during the study were recorded by the nurses and post-visit episodes determined by telephone follow-up. Heart rate and systolic and diastolic blood pressure and were measured bedside throughout each study visit.

#### Data Analysis and Statistics

Basal glucose and hormone levels and ISRs were calculated for each individual by averaging values for the -90, -75, and -60 time points for all study visits. Means, SDs, and SEMs were then calculated for each group. ISRs were derived by stochastic deconvolution of the peripheral C-peptide concentrations as in earlier studies [60,61] using population-based estimates of C-peptide clearance kinetics [62–64]. Differences in baseline characteristics and/or placebo treatments between groups were evaluated by one-way ANOVA for continuous variables and fisher exact test for categorical variables. Areas under the curve (AUCs) were calculated using the trapezoid method and incremental AUCs (iAUCs) were determined by subtracting baseline AUC from the AUC. Data for AUCs and iAUCs were analyzed using mixed effects models with subject as a random effect and bethanechol as a fixed effect using SAS v9.4. Baseline values were used as a covariate for the analysis of the AUCs but not iAUCs. Paired comparisons were limited to evaluating the effects of 50, 100, and 150 mg bethanechol compared to placebo. Outcome measures through time were analyzed using the mixed random effects repeated measures model with covariance structure estimated by a spatial model (SAS 9.4). Subject and subject by drug interaction were random effects.

### Xenin-25 Study with Archived Samples

#### Subjects

**Fig 2:**
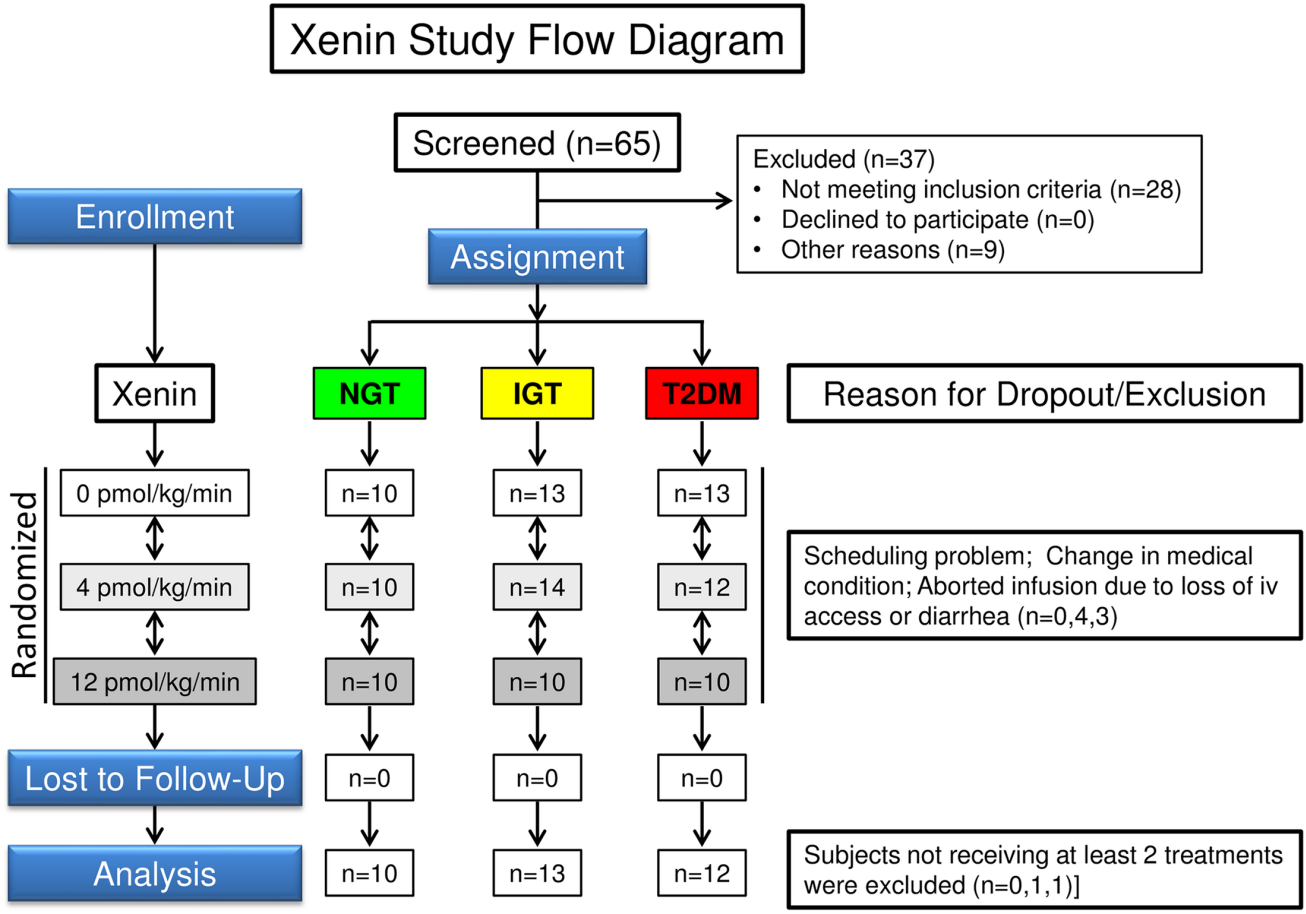
Flow diagram for xenin study. Diagram is for a non-randomized crossover study with different doses of xenin-25 in humans with NGT, IGT, and T2DM.

The study protocol was approved by Washington University’s Human Research Protection Office and the FDA (IND#103,374) and was registered with ClinicalTrials.gov (NCT00949663). Studies were performed in the Clinical Research Unit of the Institute of Clinical and Translational Sciences of Washington University after obtaining written informed consent. Study design (Fig 2), detailed procedures and an initial set of results have been published [60]. Subject recruitment was initiated on January 1, 2010 and follow-up for the final participant was completed on March 20, 2012. Subjects had been recruited through Washington University’s Volunteers for Health Office and were remunerated to encourage completion of the study. No new subjects were enrolled or studied for the present report and only a subset of archived plasma samples from the prior study were analyzed (Table 1; n = 10 per group). Patients gave written informed consent for future analyses of archived samples. There were no adverse events or unintended effects during this study.

#### Study Design

Briefly, subjects were screened, assigned to groups with NGT, IGT, and T2DM, and then studied as described above. However, instead of oral bethanechol administration at minus 60 min, a primed-constant intravenous infusion of xenin-25 (4 or 12 pmoles/kg/min) or placebo [albumin alone (Alb)] was initiated along with Boost Plus ingestion at time zero and peptide infusion was continued until the 300-min time point. PP assays and data analysis are as described above. Group characteristics in the xenin-25 study [60] were very similar to those in the bethanechol study (Table 1).

## Results

### Subject Characteristics

For the bethanechol study, values for two-hour glucose, fasting glucose and HbA1c were generally in the order of NGT<IGT<T2DM (Table 1). Body mass index, age, and gender were not statistically different between groups. Six of the 9 subjects with T2DM were treated with metformin. Of subjects with T2DM, two required insulin before one visit and two required insulin before three visits. Eight patients were withdrawn from the study because: one had difficulty swallowing the ACM, two had low baseline blood pressure measurements, two were lost to follow-up, one had anemia, one experienced hypertension during the recovery time and one had a normal OGTT but was taking metformin. Basal values for each parameter were typically similar for each individual at each of their visits but a post hoc analysis of data revealed that subject #179 (T2DM) had not fasted before the 0 mg visit. Because this subject had received the 25 mg dose of bethanechol, data for this visit were used in place of the placebo. No studies were aborted due to bradycardia (<50 beats per min), symptomatic hypotension or lightheadedness, nausea, vomiting or diarrhea. However, with the 100 mg dose of bethanechol, 2 subjects (1 each with IGT and T2DM) experienced an asymptomatic drop in mean arterial pressure and 1 subject (with IGT) experienced an asymptomatic increase in heart rate. These 3 subjects were not administered the 150 mg dose of bethanechol.

### The postprandial PP response is similar in humans with and without T2DM

**Fig 3:**
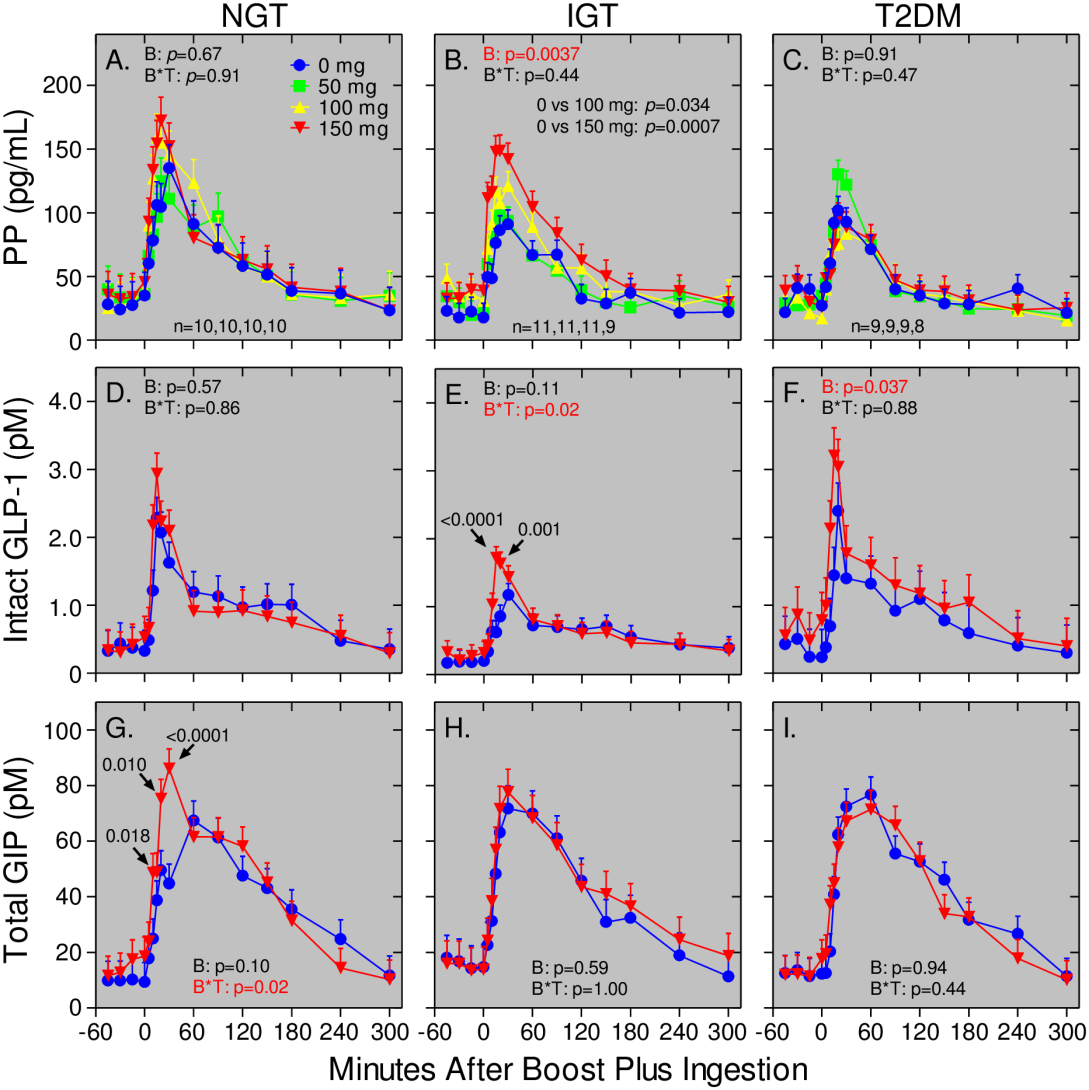
Bethanechol differentially affects PP, GLP-1, and GIP responses in humans with NGT, IGT, and T2DM. Subjects with NGT, IGT, and T2DM were administered separate meal tolerance tests with placebo (blue dots) or bethanechol at a dose of 50 mg (green squares), 100 mg (yellow triangles), or 150 mg (inverted red triangles). Plasma levels of PP (Panels A-C), intact GLP-1 (Panels D-F), and total GIP (Panels G-I) were measured at the indicated times before and after meal ingestion. Values represent group means ± SEMs for subjects with NGT (Panels A, D, G), IGT (Panels B, E, H), and T2DM (Panels C, F, I). The number of subjects receiving the 0, 50, 100, and 150 mg dose of bethanechol is indicated for each group. Differences in subject number within each group are because several subjects did not receive the 150 mg dose. GLP-1 and GIP levels were only measured in samples from individual subjects receiving both the 0 mg and 150 mg doses of bethanechol. *P* values for the bethanechol effect (B) and for bethanechol-time interaction (B*T) are indicated in each panel. Statistically significant *P* values for individual time points are shown if the bethanechol or bethanechol-time interaction was significant.

**Fig 4:**
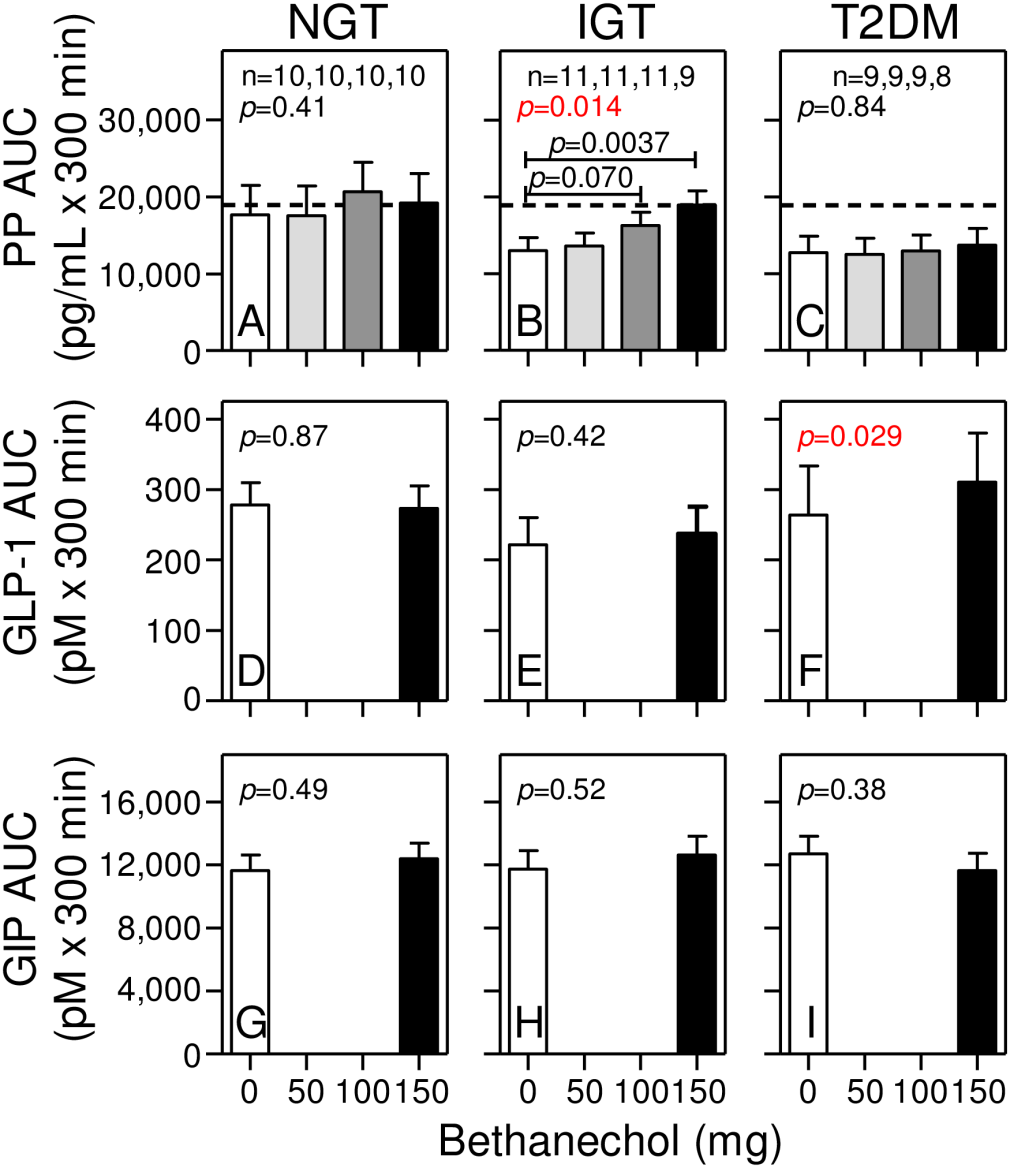
Bethanechol increases the PP AUC only in humans with IGT. Areas under the curve (AUC) were calculated for each individual at the indicated dose of bethanechol from data shown in Fig 3. Group means ± SEM are shown. *P* values for a bethanechol effect were determined using the mixed effects model and are shown in each panel. Statistically significant *p* values for each dose of bethanechol versus placebo are shown only if the bethanechol effect is significant.

**Fig 5:**
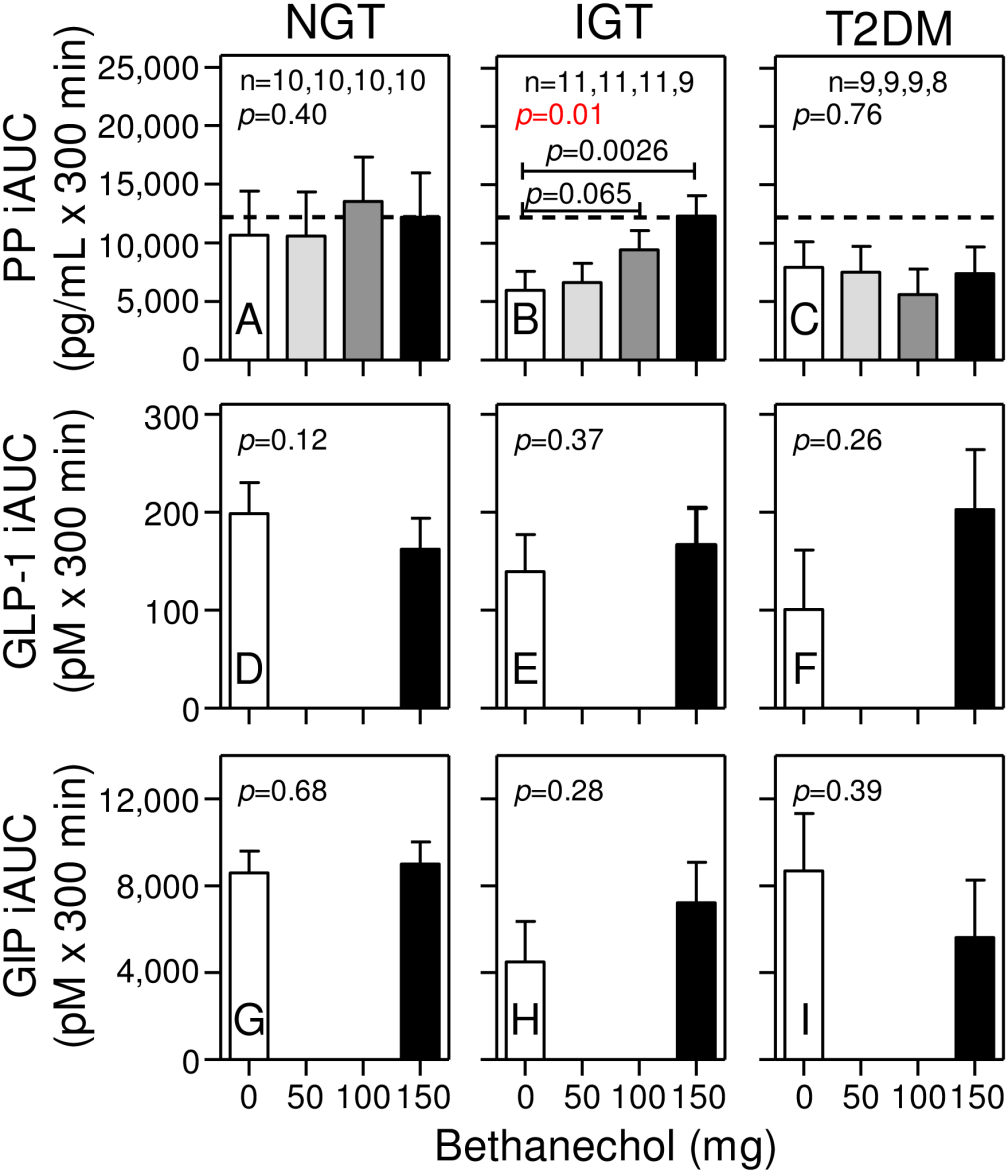
Bethanechol increases the PP iAUC only in humans with IGT. Incremental AUCs were calculated for each individual at the indicated dose of bethanechol from data shown in Fig 3. Group means ± SEM are shown. *P* values were determined using the mixed effects model. Statistics are as in Fig 4.

In the bethanechol study, fasting PP levels were not different between groups (Table 1). As shown in Fig 3A–3C, administration of the placebo (with water) caused small changes in PP levels over the next 60 min (i.e. prior to meal ingestion). With meal ingestion, levels rapidly increased in all 3 groups, peaked by 30 min and then slowly returned to baseline values by ~180 min. Group differences in the PP AUCs (Fig 4A–4C) and iAUCs (Fig 5A–5C) from 0 to 300 min in response to placebo did not reach statistical significance (*p* = 0.30 and *p* = 0.34, respectively, by 1-way ANOVA).

### Bethanechol increases the PP response in humans with IGT, but not NGT or T2DM

To determine the effects of bethanechol within each group, the meal tolerance tests were repeated with escalating doses of bethanechol, with each dose administered during a subsequent visit. Compared to placebo, ingestion of 50, 100, and 150 mg bethanechol had no statistically significant effect on PP levels before meal ingestion (Fig 3A–3C; *p* = 0.76, *p* = 0.36, and *p* = 0.35 in groups with NGT, IGT, and T2DM, respectively). However, after meal ingestion, there were progressive and bethanechol-dose-dependent increases in the PP levels (Fig 3B; *p*<0.004), AUCs (Fig 4B; *p* = 0.014), and iAUCs (Fig 5B; *p* = 0.01) in the subjects with IGT, but not NGT or T2DM (Figs 3A–3C, 4A–4C and 5A–5C). The dose-response in AUCs was linear in the IGT group (*p* = 0.0014). With the 150 mg dose versus the placebo, the AUC and iAUC in the IGT group increased 1.45-fold (*p* = 0.0037) and 2-fold (*p* = 0.0026), respectively.

### Bethanechol increases the GLP-1 response in humans with IGT and T2DM, but not NGT

**Fig 6:**
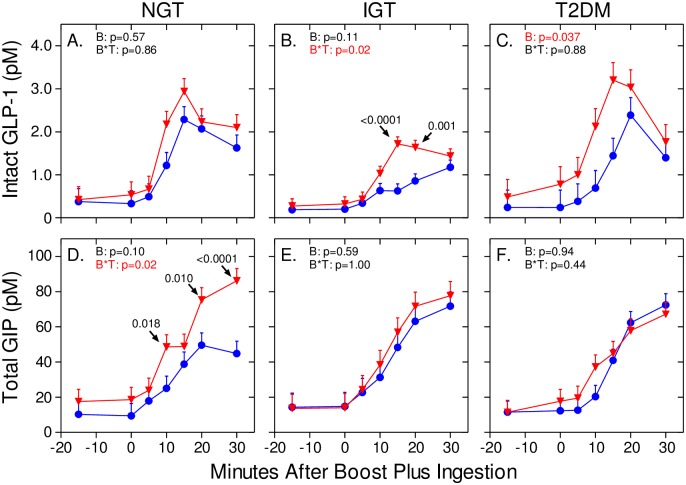
Bethanechol differentially affects GIP and GLP-1 responses in humans with NGT, IGT, and T2DM. Selected data from Fig 3 (-15 to 30 min) are expanded to emphasize differential GLP-1 (panels A-C) and GIP (panels D-F) responses to the 150 mg dose of bethanechol.

Next, intact GLP-1 levels were measured in samples from the 0 mg and 150 mg study visits. As shown in Fig 3D–3F, plasma levels of intact GLP-1 increased rapidly after meal ingestion. With placebo, levels peaked at 15–20 min, rapidly declined until 60 min, and then slowly decreased until the 300 min time point. Bethanechol slightly increased the early GLP-1 responses in the groups with NGT (Figs 3D and 6D) and IGT (Figs 3E and 6E) but this response was statistically significant only in the IGT group (*p* = 0.02 for a bethanechol-time interaction). In contrast, bethanechol increased the postprandial GLP-1 response in the group with T2DM (Figs 3F and 6F; *p* = 0.037 for bethanechol effect). The 300-min AUC for GLP-1 was also significantly increased by the 150 mg dose of bethanechol (p = 0.029; Fig 4F).

### Bethanechol increases early GIP release in humans with NGT, but not IGT or T2DM

As shown in Figs 3G–3I and 6D–4F, plasma levels of total GIP increased rapidly after meal ingestion, peaked 30 to 60 min later and then continually declined until the 300 min time point. With the placebo, postprandial peak GIP levels were similar in all 3 groups (Fig 3G–3I). However, bethanechol administration increased peak GIP levels in the group with NGT, but not IGT or T2DM. A repeated measures 2-way ANOVA revealed that the interaction between time and bethanechol was highly significant in the group with NGT (*p* = 0.02 for a bethanechol-time interaction), but not IGT (p = 1.0) or T2DM (p = 0.44). The 300-min GIP AUC (Fig 4G–4I) and iAUC (Fig 5G–5I) were not significantly altered by 150 mg bethanechol in any group.

### Bethanechol has no effect on glucose homeostasis in humans with NGT, IGT, and T2DM

**Fig 7:**
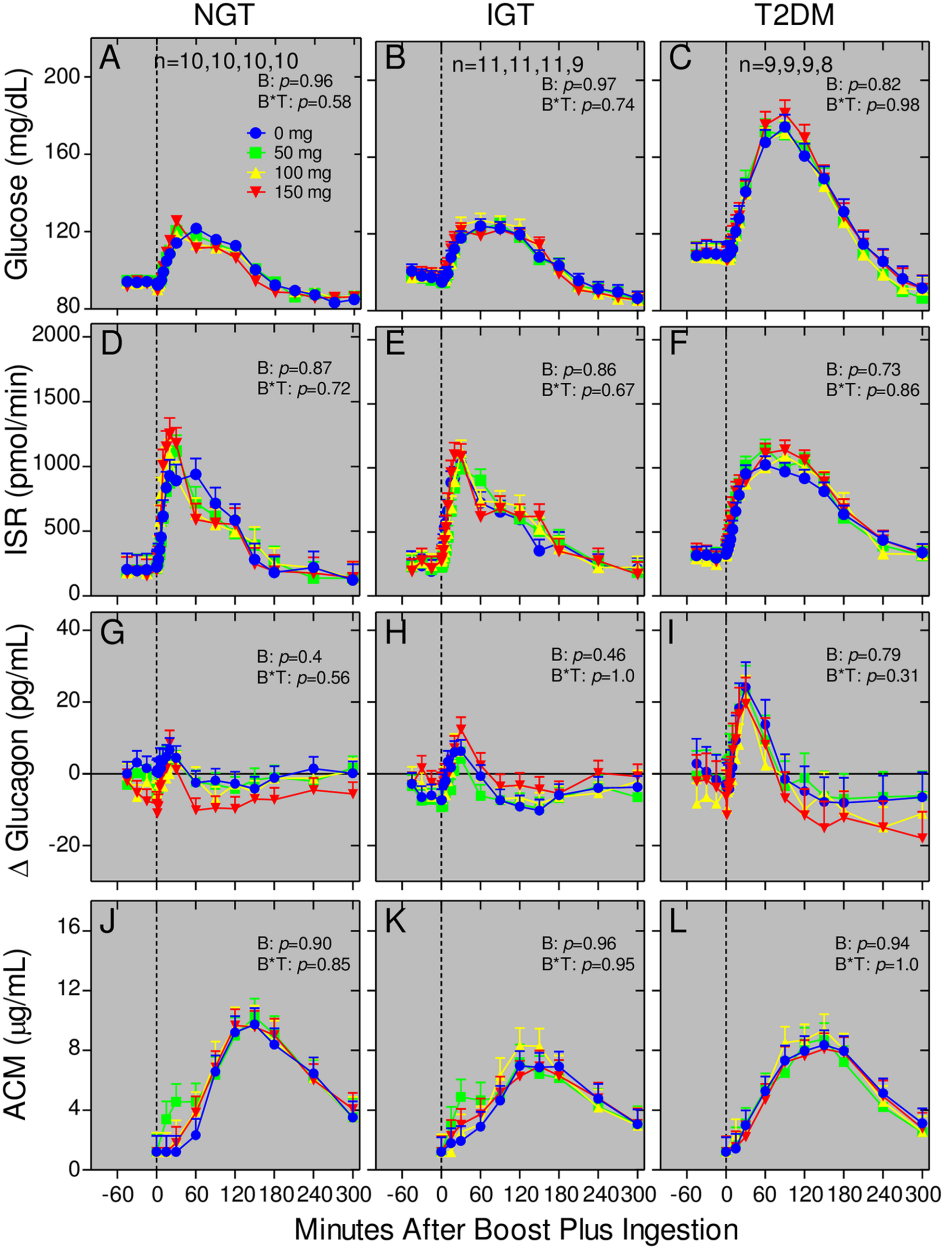
Bethanechol has no effect on glucose homeostasis in humans with NGT, IGT, and T2DM. Plasma glucose (panels A-C), glucagon (panels G-I), and ACM (panels J-L) levels and insulin secretion rates (panels D-F) were determined during meal tolerances as described in Fig 3. Values represent group means ± SEMs for subjects with NGT (Panels A, D, G, J), IGT (Panels B, E, H, K), and T2DM (Panels C, F, I, L). Symbols are the same as in Fig 4. *P* values for bethanechol and for bethanechol-time interaction are indicated in each panel. Data for the 100 mg dose of bethanechol for one subject with T2DM was excluded from the analysis because baseline values were 4.5 standard deviations from the mean.

**Fig 8:**
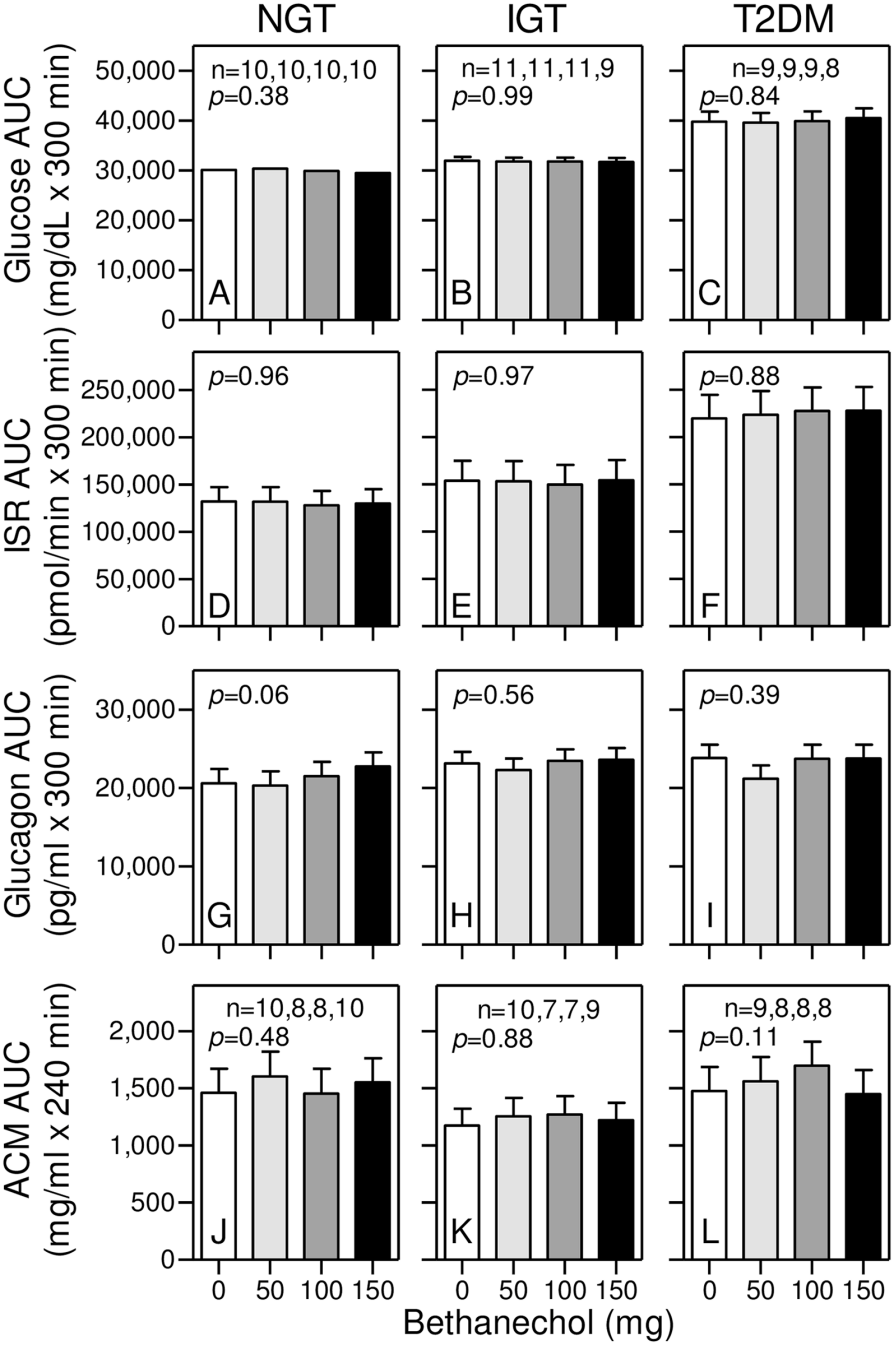
Bethanechol has no effect on glucose homeostasis (AUCs) in humans. Areas under the curves (AUCs) were calculated from data shown in Fig 7. Group means ± SEM are shown. There were no statistically significant differences in any response within each group.

**Fig 9:**
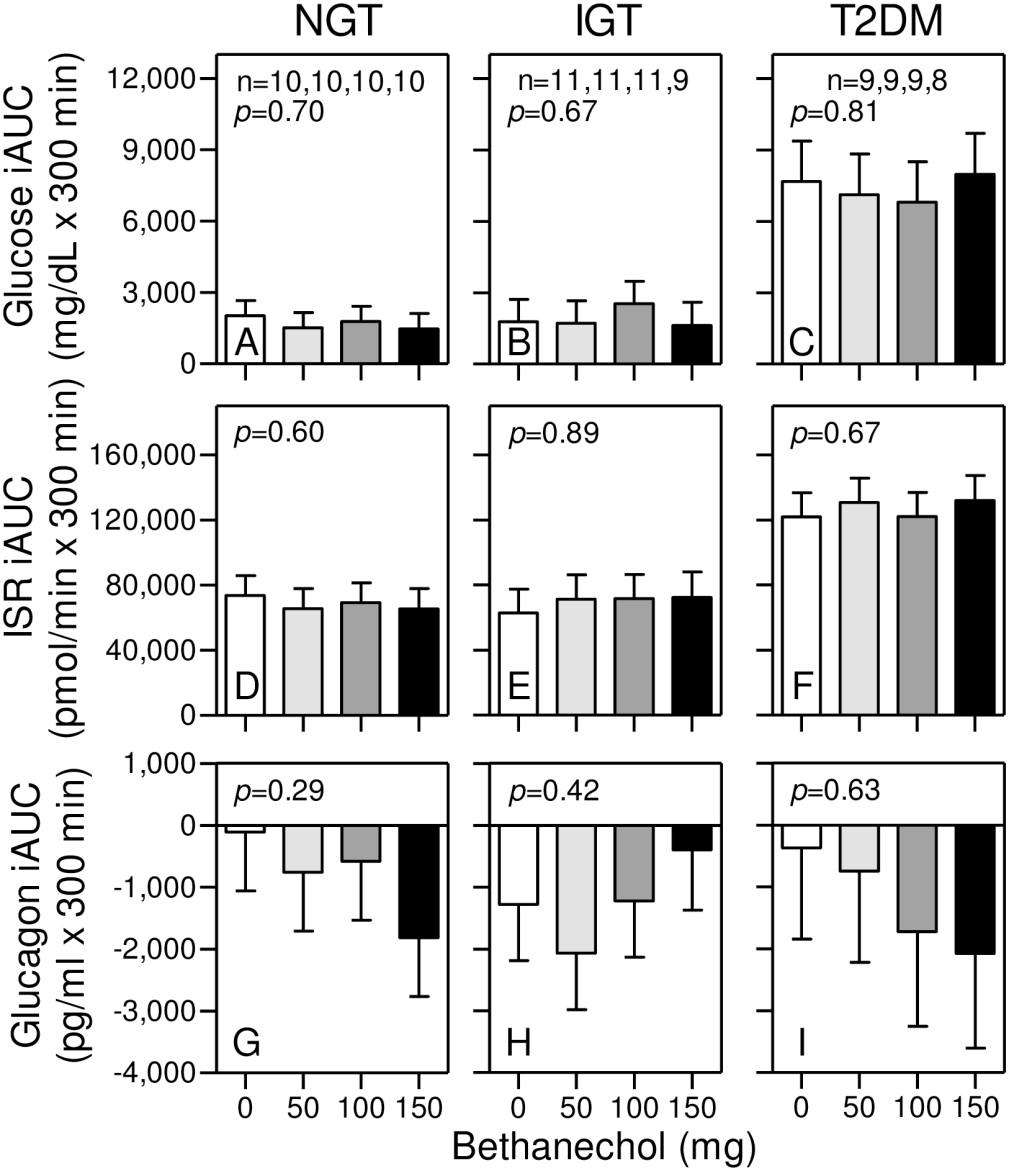
Bethanechol has no effect on glucose homeostasis (iAUCs) in humans. Incremental AUCs were calculated from data shown in Fig 7. Group means ± SEM are shown. There were no statistically significant differences in any response within each group.

Consistent with progressively worsening glucose tolerance, fasting and postprandial plasma glucose levels (Fig 7A–7C) and ISRs (Fig 7D–7F) as well as their respective AUCs (Fig 8A–8F) and iAUCS (Fig 9A–9F) with the placebo were in the order of T2DM>IGT>NGT (*p*<0.0001 and p = 0.04 for respective glucose and ISR AUCs). Moreover, there were rapid and transient increases in the postprandial glucagon responses in the order of T2DM>IGT>NGT (Fig 7G–7I) after which glucagon levels decreased (*p* = 0.05 and *p* = 0.46 for respective 0–60 and 0–300 minute AUCs). Unlike PP, GLP-1, and GIP responses, there were no statistically significant bethanechol or bethanechol-time interactions affecting glucose, ISRs, or glucagon levels (Fig 7A–7I), AUCs (Fig 8A–8I), and iAUCs (Fig 9A–9I) within any group. Similarly, bethanechol did not affect the levels, times to peak value, AUCs and iAUCs for plasma ACM (Figs 7J–7L, 8J–8L and 9J–9L) indicating that it did not affect the rate of gastric emptying.

### Symptomatic effects of bethanechol

**Fig 10:**
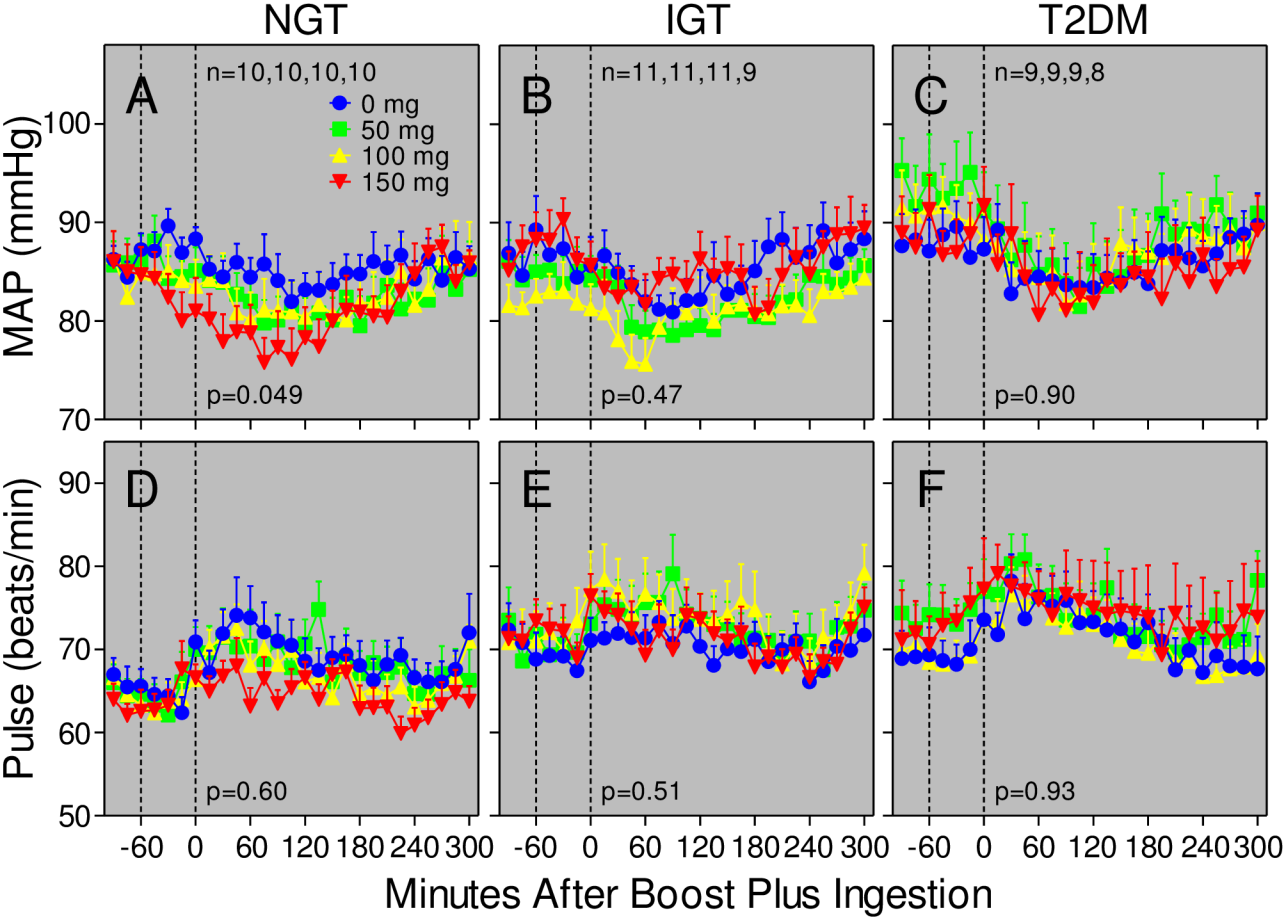
Bethanechol has little effect on blood pressure or heart rate in humans. Mean arterial blood pressure (MAP) was calculated from systolic (SP) and diastolic (DP) blood pressure readings using the formula MAP = DP+1/3(SP-DP). Values represent group means ± SEMs for subjects with NGT (Panels A, D), IGT (Panels B, E), and T2DM (Panels C, F). Symbols and statistics for bethanechol-time interaction are as described in Fig 3.

Subject surveys indicated that there were no dose-dependent effects of bethanechol on diarrhea, nausea, vomiting, chest pains, dizziness, heart palpitations, shortness of breath, fever, chills, blurred vision, or changes in salivation, sweating, or frequency of urination. Meal ingestion transiently reduced the group mean arterial blood pressure (7–10 mmHg) and increased resting heart rate (~8 beats per min) in each group but bethanechol had little additional dose-dependent effect on either outcome (Fig 10). The absence of significant effects on sweating, blood pressure, and heart rate are consistent with previous studies by others administering similar doses of oral bethanechol [55,56].

### Xenin-25 infusion increases the PP response in humans with NGT, IGT, and T2DM

**Fig 11:**
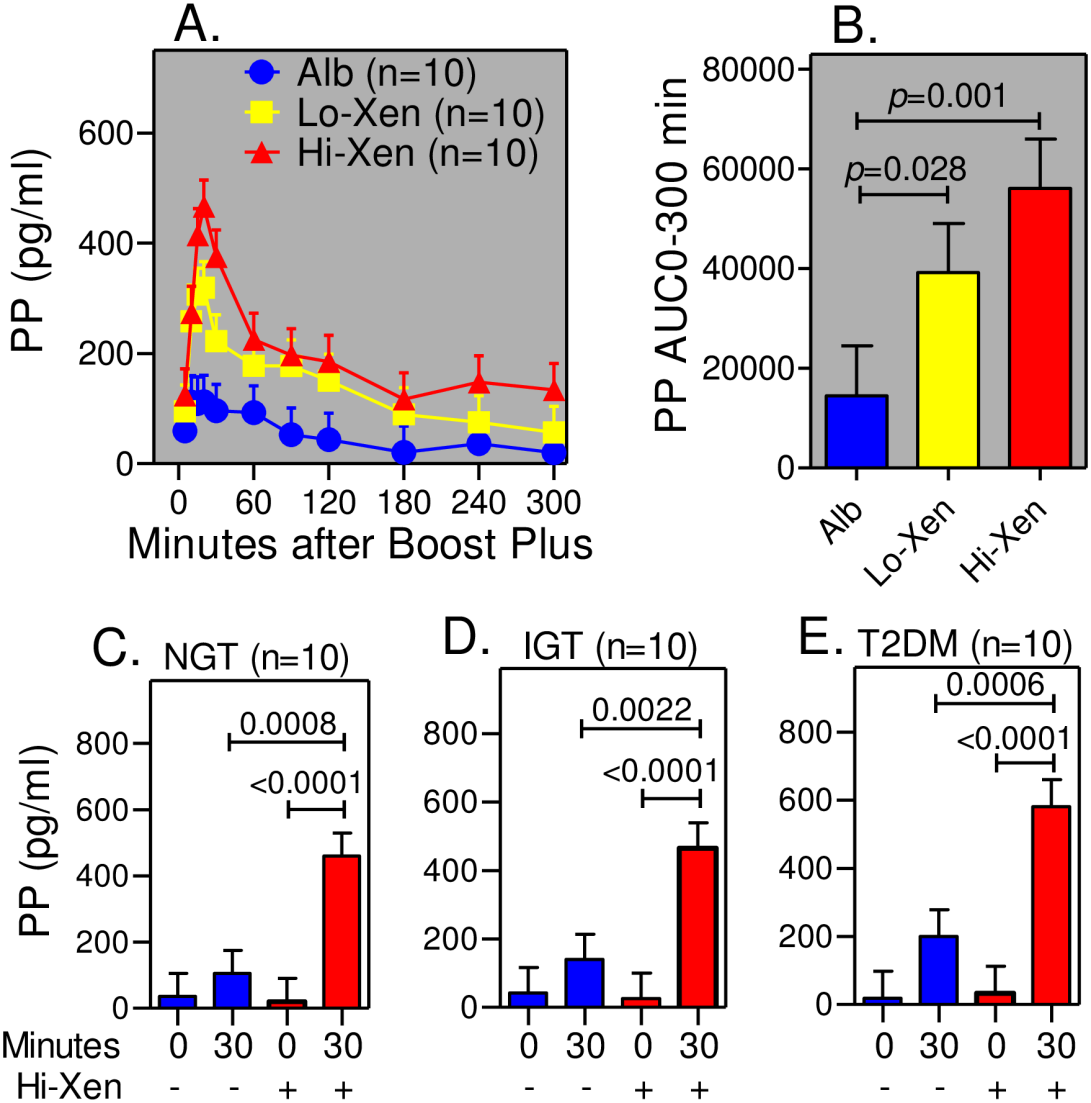
Xenin-25 profoundly increases PP release in humans with NG, IGT, and T2DM. (A) PP levels were measured at the indicated times in subjects with NGT during meal tolerance tests with albumin alone (Alb; blue dots) or xenin-25 at a dose of 4 pmol/kg/min (Lo-Xen; yellow squares) or 12 pmol/kg/min (Hi-Xen; red triangles). As in the bethanechol study, the liquid meal was ingested from 0–3 min. (B) 0–300 min AUCs for data in panel A are shown. (C-E) PP levels were measured in the samples collected before (0 min) and 30 min after meal ingestion in humans with NGT (Panel C), IGT (Panel D), and T2DM (Panel E) during a primed-continuous infusion of xenin-25 (12 pmoles/kg/min).

We previously showed that intravenous administration of an intestinal peptide called xenin-25 during intravenous graded glucose infusions increases the PP response in humans with NGT, IGT, and T2DM [33]. This PP response is completely inhibited by atropine sulfate (manuscript in preparation) indicating it is mediated by increased cholinergic input to islets. Thus, we determined if xenin-25 also amplifies postprandial PP responses and if these responses are larger than those measured after administration of 150 mg bethanechol. Archived plasma samples from our previous xenin study were used for these measurements [60]. A detailed examination of the PP response in humans with NGT showed that basal PP concentrations and postprandial levels, temporal profiles, and 300-min AUCs were similar to those measured during placebo administration in the bethanechol study (Not Shown; 300-min PP AUCs = 15,747 ± 2800 vs 17,705 ± 4476, respectively). Hence, timing and route of drug administration does not affect the PP response. Further, the PP response was increased by xenin-25 infusion in a dose-dependent fashion (Fig 11A and 11B). Because PP levels peak ~30 min after meal ingestion, PP levels were measured in additional samples from subjects with IGT and T2DM prepared 30 min after boost plus ingestion and compared to those from the bethanechol study (150 mg dose). As shown in Fig 11C–11E, infusion of xenin-25 increased PP levels to a much greater extent than the highest dose of bethanechol in subjects with NGT, IGT, and T2DM (Fig 3A–3C). However, infusion of xenin had no effect on postprandial ISRs in any group when normalized to plasma glucose levels [60].

## Discussion

Bethanechol does not cross the blood-brain barrier and thus, can be used to study the effects of muscarinic cholinergic signaling in the periphery on glucose homeostasis. The PP response was used as a surrogate measure for cholinergic input to islets. Results showed that PP levels, AUCs, and iAUCs were not different between groups during administration of the placebo. Bethanechol had relatively little effect on PP levels before meal ingestion but significantly amplified postprandial PP levels, AUCs (1.45-fold), and iAUCs (2-fold) in humans with IGT in a dose-dependent fashion. In contrast, bethanechol did not increase the PP response in the groups with NGT and T2DM. Thus, PP cells in humans with IGT, but not NGT or T2DM, develop hypersensitivity to cholinergic input. Even though the highest dose of bethanechol increased the PP response in the IGT group, there was no corresponding effect on plasma glucose or glucagon concentrations, ISRs, or gastric emptying. In contrast to bethanechol, infusion of xenin-25 at 12 pmoles/kg/min increased the PP response nearly 4-fold in all 3 groups. As we previously reported, this dose of xenin-25 delayed gastric emptying but did not affect ISRs when normalized to plasma glucose levels [60]. These collective results suggest that increasing cholinergic input to islets plays only a minor role in regulating postprandial insulin and glucagon secretion in humans.

Consistent with our earlier [33] and present (Figs 3, 4 and 5) studies, others have also reported that PP levels and responses are not increased in humans with T2DM [65,66]. In contrast, some studies concluded that PP levels and responses are increased in T2DM [67,68]. However, PP levels and responses are known to dramatically increase with age [65,69] and protein and fat elicit much larger PP responses compared to oral glucose [65]. Additionally, in the present study an extraction step was incorporated to remove contaminants that artifactually increase PP levels in human plasma samples [33]. Further, a dual-antibody sandwich ELISA that does not cross react with highly related NPY and PYY or with other gut peptides was used for our PP measurements. This type of ELISA exhibits greater antigen specificity than that obtained using a single antibody RIA. Hence, the reason(s) for the discrepancies in PP responses in humans with versus without T2DM is likely due to differences in assay procedures and specificities but also raises the intriguing possibility that propancreatic polypeptide may be differentially processed to peptides with unique bioactivities in humans with NGT, IGT, and T2DM.

An unexpected finding was that bethanechol had complex and differential effects on GLP-1 and GIP release in humans with NGT, IGT, and T2DM during mixed meal tolerance tests. Specifically, bethanechol increased the GLP-1 response in the groups with IGT and T2DM (T2DM > IGT) but increased GIP release only in the group with NGT. Our study was not designed to determine if these were direct or indirect effects of bethanechol action on intestinal K and/or L cells. In spite of this, changes in the patterns for GIP and GLP-1 release were not accompanied by alterations in profiles for ISRs, rate of gastric emptying, plasma glucagon levels, and glucose concentrations within any group. This is consistent with previous results from our laboratory showing that postprandial, endogenously released, circulating GLP-1 plays little role in regulating postprandial insulin secretion in humans [60]. That bethanechol altered GLP-1 release but not insulin secretion in the group with T2DM is also consistent with the well-known observation that the response to endogenous incretins is blunted in T2DM [70]. Thus, strategies to increase release of endogenous GLP-1 in humans with T2DM may not represent an effective intervention for treating this disease. It is also important to note that the GLP-1 response is as rapid as that for GIP even though most GLP-1 producing cells are located in the distal intestine whereas GIP-producing cells reside predominantly in the proximal gut. This suggests that early GLP-1 release is mainly under neural rather than nutritional control. Consistent with this idea, a host of neurotransmitters and peptides increase GLP-1 release in the vascularly perfused rat ileum [71].

Several limitations to the current study should be addressed. First, bethanechol had only modest effects on the PP, GIP, and GLP-1 responses within each effected group. Although the doses used in our study are known to affect the stretch response of the bladder muscle without eliciting hypotension or bradycardia [55,56], it is possible that higher doses would have exerted greater effects and thus, possibly altered glucose levels, ISRs, glucagon concentrations, gastric emptying, or other factors that regulate glucose homeostasis. However, the bethanechol doses used in the current study revealed that physiologically relevant changes in endogenous PP, GIP, and GLP-1 release do not affect postprandial glucose homeostasis. This result could have potentially been masked by a greater degree of cholinergic agonism. However, that xenin-25 profoundly increased PP release but not ISRs argues against this. A second limitation is that bethanechol activates all muscarinic receptors in the periphery and thus, the current study assessed the effects of stimulating multiple superimposed cholinergic signaling pathways and it is possible that numerous positive and negative responses exactly offset each other. If this is the case, it would suggest that cholinergic signaling could possibly act to maintain a pre-established “set-point” for glucose homeostasis without increasing insulin secretion. It should be noted that of the 5 known muscarinic acetylcholine receptors, it is the M3 subtype that increases insulin release from beta cells [7–12]. Thus, even though a xenin-25-mediated increase in cholinergic input to islets did not amplify ISRs, M3 subtype-specific agonists may still represent a therapeutic strategy for increasing ISRs in T2DM. Third, the lower doses of bethanechol may not have remained active for the duration of the study though it is clear that the 150 mg dose had effects in all groups and the PP response in the group with IGT was bethanechol dose-dependent. Finally, our results compared the effects of cholinergic signaling on postprandial ISRs and glucagon levels in humans with NGT, IGT, and T2DM. However, our results may not be applicable to other metabolic or pathophysiologic conditions.

## Conclusions

In spite of the limitations, our results indicate that bethanechol has different effects on PP, GIP, and GLP-1 release in humans with NGT versus IGT versus T2DM. Even with these differences, bethanechol had no measureable effect on glucose homeostasis in any group. As shown in this as well as our earlier study [33], infusion of xenin-25 in humans with NGT increased the postprandial PP response nearly 4-fold and reduced the GLP-1 response 6-fold but had no effect on insulin or glucagon responses. These results suggest that cholinergic signaling and circulating GLP-1 play only minor roles in regulating glucose homeostasis in humans. Because islet responses to endogenously released circulating incretins [70] but not to exogenously infused GIP [61] or GLP-1 [72,73] are blunted in T2DM, it is critical to determine if and how GLP-1 and GIP released from respective intestinal L and K cells regulate glucose homeostasis in humans. Moreover, our data suggest that cholinergic mechanisms that regulate insulin secretion are different in humans and mice and thus, extreme caution must be exercised when extrapolating results from animals to humans.

## Supporting Information

S1 Appendix Original IRB approved protocol for bethanechol study. (PDF)

S2 Appendix Trend Statement for bethanechol study. (PDF)
