# Supplementary material for: Hormonal Responses to Cholinergic Input Are Different in Humans with and without Type 2 Diabetes Mellitus
Source: PLoS One. 2016 Jun 15;11(6):e0156852. doi: 10.1371/journal.pone.0156852 (PMC4909255; doi:10.1371/journal.pone.0156852)
Supplement: S1 Appendix — (PDF) [file pone.0156852.s001.pdf]

Feb 02, 2011

Titles:
1) Restoration of the GIP-mediated incretin effect in persons with type 2 diabetes mellitus
2) The role of Xenin-25 in mediating GIP action in humans with type 2 diabetes
3) A proof-of-concept clinical trial of Xenin-25 in the treatment of type 2 Diabetes in humans
4) The effects of xenin-25 on GIP action in humans with type 2 diabetes
5) Xenin-25: novel regulator of insulin secretion and β-cell function

Co-Principal Investigators:

Burton M. Wice, Ph.D.
Research Associate Professor of Medicine
Department of Internal Medicine
Division of Endocrinology, Metabolism, and Lipid Research

Dominic Reeds, M.D.
Assistant Professor Geriatrics and Nutritional Science
Department of Internal Medicine – Nutritional Science

Co-Investigators:

Judit Dunai, M.D.
Clinical Fellow
Department of Internal Medicine– Endocrinology/Metabolism

Bruce Patterson, Ph.D.
Research Associate Professor of Medicine
Division of Nutritional Science

Research Team Members: PIs, Co-Investigators as listed above, David Rometo, M.D. and Erin Laciny, MSEd.

<u>Research Design and Methods:</u>

All studies will be performed in the Clinical Research Unit (CRU) of the Institute of Clinical and Translational Sciences of Washington University after written informed consent has been obtained. Dr. Wice is not a physician and thus, will not be involved in making any medical decisions concerning patient safety and/or treatment. His primary role will be to ensure that the study protocols are consistent with those described in the research grants and approved by the FDA under IND #103,374. All medical decisions concerning patient safety and/or treatment will be made by Dr. Reeds. In addition to the actual Study Coordinator, Those authorized to obtain consent are: Dominic Reeds, M.D., Judit Dunai, M.D., David Rometo, M.D. Kristin Kraus, RN, CRC and Erin Laciny, MSEd. General inclusion/exclusion criteria will be the same for all Protocols are listed below and any Protocol-specific changes to these criteria are noted in the appropriate Protocol. Each Protocol will be conducted using subjects that exhibit normal glucose tolerance (NGT), impaired glucose tolerance (IGT), and mild T2DM (15 subjects per group). Glucose tolerance will be defined by the 2-hour glucose level noted during the screening OGTT using the ADA Expert Committee recommendations for diagnostic criteria as noted below.

- NGT: 2-hr glucose result < 140mg/dl.
- IGT: 2 hr glucose result 140-199 mg/dl.
- T2DM: 2 hour reading ≥200mg/dl OR a fasting glucometer reading of ≥150mg/dl. If the fasting glucose level is 150 mg/dl or greater, that subject will automatically be enrolled into the diabetic group with confirmation of enrollment after screening labs have been reviewed & meet study criteria.

Inclusion Criteria:

- Ages 18-65.
- Individuals must be able to consent for their own participation (no mental impairment affecting cognition or willingness to follow study instructions).
- Healthy volunteers with no clinical evidence of T2DM (see below).
- Otherwise healthy volunteers that have impaired glucose tolerance (see below).
- Otherwise healthy volunteers with Diet Controlled T2DM (see below).
- Otherwise healthy volunteers withT2DM that take oral agents only if the subject's pre-existing oral anti-diabetic agents can be safely discontinued for 48-hours (fasting glucose level remains <150mg/dl in order to do the OGTT).
- Otherwise healthy volunteers with T2DM who do not use insulin for blood glucose control.
- Persons with HbA1c ≤ 9%.
- Women of childbearing potential must be currently taking/using a method of birth control that is acceptable to the investigators. A pregnancy test will be done at the beginning of each visit. Any woman with a positive pregnancy test will be removed from the study.
- Willingness to return have 8-10ml of blood drawn 25-30 days after the last Xenin infusion; to check for Xenin peptide antibodies that MAY develop. (All efforts will be made to complete this visit during study participation.)

Exclusion Criteria:

- <18years of age or >65 years of age.
- Lacks cognitive ability to sign the consent &/or follow the study directions for themselves.
- Women unwilling to comply with using an acceptable method of contraception during the course of the study, or who are currently breast-feeding.
- Any subject whose screening HbA1c is >9.0%.
- Type 2 diabetes requiring the use of supplemental insulin @ home.
- Volunteers with a history of Acute Pancreatitis.
- Volunteers with a history of cancer (except for skin cancer).
- Volunteer with a history of Chronic Pancreatitis and/or risk factors for chronic pancreatitis including hypertriglyceridemia (triglycerides >400mg/ml) hypercalcemia (blood calcium level >11.md/dl) and/or the presence of gallstones.
- Volunteers with a history of gastrointestinal disorders, particularly related to gastric motility/emptying such as gastric bypass, documented gastro-paresis in diabetic volunteers.
- Subjects taking medications known to affect glucose tolerance.
- Hematocrit from the lab is below 33% (or if the finger stick hemoglobin measured with the HemoCue 201+ is <11.2%g/dl).
- Diabetics that have the potential to have a low blood sugar without them being aware that their blood sugar is low (hypoglycemia unawareness).
- Any major medical conditions, or conditions that in the opinion of the PI make the subject unsuitable for the study.
- Subjects with abnormal kidney function as measured by the Creatinine concentration will be excluded.

- Subjects with a history of active liver disease or AST/ALT levels >2X upper limit of normal will also be excluded.
- Total Bilirubin levels should be <2.
- Subjects unwilling to allow the use of their own blood or albumin in the preparation of the peptides. (The blood will prevent sticking of the peptide to the tubing; an alternative method has been sought but not found.)
- Unwillingness to allow blood glucose level adjustment (if needed) with IV insulin
- Unwillingness to return have 8-10ml of blood drawn 25-30 days after the last Xenin infusion; to check for Xenin peptide antibodies that MAY develop. (All efforts will be made to complete this visit during study participation.)

SELECTION OF SUBJECTS: Studies will be conducted with subjects who meet the above inclusion criteria. Subjects with impaired glucose tolerance or T2DM will be compared with control subjects with normal glucose tolerance, with group characteristics similar to the extent possible with respect to age, sex, race and degree of obesity as judged by the body mass index (BMI). In view of the importance of body fat distribution in the determination of insulin sensitivity and glucose tolerance (1-3), all groups will be matched for this variable to the extent possible. We may perform DEXA body fat composition study scans on all patients. (DXA – Hologic Discovery, weight limit 300lbs or >136kg. For those subjects that exceed this limit, the GE Lunar machine may be used as it has a weight limit of 450lbs or 205kg.) This will involve exposure to 6 microseverts (or the equivalent of a 1.4mGy dose maximum) of radiation (less than the amount one would be exposed to when flying across the continental U.S. and significantly less than that of a chest x-ray). Because of possible variation in insulin secretion at different phases of the menstrual cycle, menstruating women will be studied, whenever possible, in the early follicular phase, unless contraceptives such as Depo-Provera, the "Pill", "-Patch" or "Ring" are in use. The first day of the last menstrual period will be noted in the Health History Questionnaire. Subjects will not be excluded on the basis of race or gender.

SCREENING: Glucose tolerance will be determined during a screening 75-g 2-hour Oral Glucose Tolerance Test (OGTT) using American Diabetes Association (ADA) Expert Committee criteria (4) during the initial screening visit (described below). Subjects with T2DM AND taking oral medications will be given the OGTT on a date following the consent visit if they meet the inclusion criteria for the study. A health history questionnaire will be performed during the screening visit. Screening laboratories will include A1C, CBC, CMP, amylase, & Lipid Panel. Participants will be asked to refrain from donating blood during this same time frame. As mentioned above; we plan to recruit up to 160 subjects in order to achieve up to 15 subjects in each group for each Protocol.

We feel that increasing the total number of subjects to 160 will adequately allow for screening failures and incomplete study (i.e., subject withdrew or was withdrawn by the PI).

EXPERIMENTAL PROTOCOLS

All protocols will be performed after a 10-12 hour overnight fast. One intravenous catheter will be placed in a suitable vein for blood withdrawal. The patency of the line will be maintained with a Normal Saline solution (0.9%) at a “keep open” or KVO rate. The affected IV site will be kept in a warming device (50-55^{0}C) to facilitate venous sampling and provide arterialized venous blood. For experiments in which test solutions will be administered, a second intravenous line will be inserted into the antecubital or suitable vein for administration of glucose and peptides. A maximum of 550 mL blood will be collected from any individual within 8 consecutive weeks. GMP grade peptides [Xenin-25, GIP, and GLP-1 (7-36 amide)] will be purchased from Bachem USA and prepared for infusion by the Washington University GMP Core Facility and/or research team members in the IV prep room. To prevent non-specific sticking to vessels and tubing, all peptide solutions will be prepared in normal saline (0.9% sodium chloride) containing 1% human serum albumin.

LABORATORY

Plasma glucose will be measured with the glucose oxidase technique using a YSI analyzer (Yellow Springs Instruments, Yellow Springs, OH). This assay has a CV of less than 2%. Plasma insulin, C-peptide, and glucagon will be measured by commercially available double antibody radioimmunoassays and/or ELISAs (Millipore, St. Charles, MO). GLP-1 (7-36 amide) and total GIP (1-42) will be measured using commercially available ELISAs (Millipore). Xenin-25 will be measured in plasma using a custom ELISA being prepared in our laboratory. Preliminary studies indicate that this assay is capable of detecting Xenin-25 in plasma at concentrations (20-150 pM) previously reported for this peptide in human plasma (5). The antibodies that we generated for this ELISA do not appear to cross react with neurotensin.

<u>Screening 2-Hour Oral Glucose Tolerance Test:</u>

<u>Procedure.</u> Baseline samples for glucose, insulin, C-peptide, glucagon, GLP-1, GIP, and Xenin-25 will be taken at times -15 and 0 min. Glucose (75g) will be ingested orally, and blood samples will be collected for glucose and hormone concentrations with the following sampling schedule: -15, 0, 30, 60, 90, and 120 minutes. All samples should be drawn within +/- 30 seconds of time point to ensure accuracy. Actual time drawn should be noted on the flow sheet. Estimated blood loss for the 2-hour OGTT is approximately 24mL.

We are requesting permission to obtain blood samples for DNA (to be drawn during the screening visits. Recent data indicate that the insulin response to a variety of secretagogues may be genetically determined. In this protocol we are particularly interested in the insulin secretory to the combination of GIP plus Xenin-25. To study this question we will determine if the insulin secretory response to GIP plus Xenin-25 is correlated with polymorphisms in various genes known to determine the insulin secretory response e.g. the GIP receptor, Kir6.2, & other ion channel genes etc. This section was moved from Protocol 1 to the screening visit here since we are requesting permission to collect a DNA sample for all participants in each study.

<u>Data Interpretation.</u> Glucose tolerance will be determined using the revised criteria of the ADA Expert Committee (4). Summary measures will be the fasting and 2-hour post load glucose values and indices of beta cell function obtained from the minimum model of C-peptide kinetics and secretion, originally proposed to describe C-peptide data during an intravenous glucose tolerance test (6) and now extended to oral glucose tolerance test data analysis (7). All glucose and C-peptide minimal model analyses will be performed using the software program SAAM II (8). It is well established that insulin, C-peptide, GLP-1 and GIP are released into the bloodstream immediately after ingestion of glucose. Concomitantly, glucagon secretion is diminished.

However, the profile of Xenin-25 release has been reported in only a single study using 8 healthy volunteers over a 45-minute post-prandial period. We are developing and validating an immunoassay that is specific for Xenin-25. This assay will be used to study Xenin-25 levels in healthy humans and importantly, define for the first time circulating Xenin-25 levels in T2DM.

After successful screening, subjects will participate in one of following protocols as detailed below Since the new Protocol 9 (Bethanechol Study) does not involve infusion of xenin-25, subjects who participated in previous Protocols can be enrolled in this study. The primary hypothesis that we will address will be to determine whether the combination of GIP plus Xenin-25 produces a greater insulin secretory response (by at least 100%) in persons with type 2 diabetes compared to the infusion of GIP alone. We have performed power calculations based on published studies in the literature of the response to exogenous GIP administration in normal and diabetic subjects. The computations were based on a target sample size of 10 subjects per group (i.e. 10 normal subjects and 10 subjects with T2DM) and on two-sided tests at the 0.05 level of significance. In all cases, we used the largest standard deviation (SD) among those that are the basis for our projected values in the groups being compared. Based on a review of the literature, GIP infusion increased the insulin secretory response to glucose up to 400% in normal subjects compared to that produced by glucose alone. In persons with T2DM, GIP did not increase insulin secretion more than glucose alone. For the purposes of these power calculations we assumed that administration of GIP plus Xenin-25 in persons with T2DM will increase the insulin secretory response to glucose by 200%. Accordingly, based on the highest SD from all these data, the power to detect this difference in the means (changes in response to GIP alone vs. GIP plus Xenin-25) will be >1.0. If administration of GIP plus Xenin-25 increases the insulin secretory response to glucose by only 100%, the power to detect significant differences will still be as high as 0.95. Based on these calculations, studying 10 individuals per group should be sufficient to test our hypothesis in each of the following protocols.

Protocol 2: To determine if xenin-25 can increase the action of endogenously released, rather than exogenously administered, GIP.

Preliminary results using Protocol 1 suggest that Xenin-25 potentiates the incretin action of exogenously administered GIP. Since GIP release, but not action, is preserved in humans with diabetes, we now propose to determine whether Xenin-25 can also potentiate the insulin secretory response to endogenously released GIP. The peptides will be infused as noted above in Protocol 1. However, in lieu of the IV D20W infusion, subjects will be asked to ingest Boost Plus (Nestle Corporation) which is a high caloric, liquid meal. Blood will be collected at -30, -20, -10, 0, 1, 3, 5, 7,10, 15, 20, 30, 60, 90, 120, 150, 180, 240 & 300 minutes after ingestion of the Boost Plus and plasma prepared for measurement of Glucose, Insulin, C-peptide, Glucagon, GIP, GLP-1 & Xenin-25 as well as other gut derived hormones (as they become measureable). Seventeen samples will require 9 ml (2 tsp) of blood. An additional 2 samples will require 12 mL of blood since CMP and amylase will also be determined on these samples. Two additional YSI blood glucose levels will be measured at 210 and 270 minutes into the infusion to ensure subjects are not becoming hypoglycemic. Total blood drawn for this visit is approximately 185 ml of blood. This includes several (if needed) 6 x 1ml amounts of blood for "recovery amounts of blood glucose samples (YSI).

3/26/2010: Intestinal peptides are known to regulate gut motility. Thus, it is important to determine the effects of xenin-25 on gastric emptying during our infusions. To accomplish this, subjects will be given 1.5 gm of acetaminophen (Tylenol) at the time of the Boost PLUS ingestion (time point 0). Plasma acetaminophen will be determined using previously scheduled samples and time points and additional volumes of blood will not be required. For accuracy in dose administration and plasma sampling, subjects will be asked to refrain from taking acetaminophen or medications containing acetaminophen for 24 hours prior to each study visit.

6/24/10:Data collection with Protocol 2 as described above has been completed in 30 subjects (10 each with NGT, IGT, and T2DM). Preliminary results suggest that as was observed with study Protocol 1, xenin-25 can safely amplify endogenous GIP-mediated insulin release in subjects with IGT but not T2DM. However, these studies were conducted with a single dose of xenin-25 (4 pmoles/kg/min) and it is possible that a higher dose of xenin-25 will potentiate the effects of endogenously released GIP in subjects with T2DM and exert a greater effect in the subjects with NGT and/or IGT. We are requesting permission to conduct one additional infusion with xenin-25 in subjects in study Protocol 2. These subjects would receive xenin-25 (without GIP) at a dose of 12 pmoles/kg/min. We have already received approval from both the FDA and HRPO to infuse combinations of GIP (0, 1.3 and 4 pmoles/kg/min) along with xenin-25 at doses of 1.3, 4, and 12 pmoles/kg/min. Thus, this additional infusion will provide important dosing information and these subjects will not be exposed to any risks that have not previously been addressed. It is hoped that this additional infusion will be conducted with newly enrolled subjects as well as with subjects who have already completed the previous infusions. Therefore, we hope to complete this study with the previously proposed number of subjects.

9/1/10: Imodium 2 mg po prn x1 may be given as needed for diarrhea. May repeat x1. It is possible that a dose of xenin-25 that is lower than the original dose is sufficient to amplify GIP action. Thus, we are also requesting permission to conduct one additional infusion with xenin-25 in subjects in study Protocol 2. Subjects will receive xenin-25 (without GIP) at a dose of 1.3 pmoles/kg/min which is 3 times lower than the dose we have safely administered to 23 subjects using this Protocol and 9 times lower than the highest dose we are currently approved to deliver. It is hoped that this additional infusion will be conducted with newly enrolled subjects as well as with subjects who have already completed the previous infusions.

2/02/11: Results with 30 subjects to date indicate that xenin-25 improves glucose homeostasis for the first 90 minutes after ingestion of the meal. This was observed in subjects with NGT, IGT, and T2DM. However, this beneficial response is transient and plasma glucose levels are slightly elevated beginning 120 minutes after meal ingestion. Thus, we wish to conduct 2 additional infusions with the higher dose of xenin-25 alone (12 pmoles/kg/min) for 30-minutes (visit 8 below) or 90-minutes (visit 9 below), after ingestion of the meal. If the beneficial effects of xenin-25 are only exerted during the first 30-90 minutes, then hyperglycemia should remain lower during this time and not worsen at the later times. Since we are greatly shortening the duration of xenin-25 infusion, these studies are expected to pose fewer potential side effects compared to the 5-hour infusions previously conducted. Since control infusions with albumin alone were conducted for 5-hours, albumin alone will be infused after the xenin-25 infusions are terminated.

Protocol 9 is designed to determine if activation of muscarinic receptors in the periphery mimics the effects of xenin-25 to increase insulin and glucagon release and if this response is perturbed as subjects progress from NGT to IGT to T2DM. Preliminary results from Protocol 2 indicate that infusion of xenin-25 at the dose of 12 pmoles/kg/min profoundly increased post-prandial plasma glucagon levels as well as insulin secretion rates in humans with T2DM, but not NGT or IGT. Since glucagon counteracts the effects of insulin, this observation unmasks a novel and important role for xenin-25-responsive enteric neurons in the pathogenesis of T2DM in humans. Therefore, it is important to determine whether acetylcholine or other neurotransmitters released from enteric neurons relay the effects of xenin-25. Bethanechol is a fast acting, short-lived acetylcholine analog that does not cross the blood-brain barrier. Thus, it activates only muscarinic receptors in the periphery. Since xenin-25 increases acetylcholine release, bethanechol bypasses the requirement for activation of xenin-25-responsive enteric neurons. If Bethanechol increases post-prandial glucagon release in humans with T2DM, then xenin-25 is working by stimulating acetylcholine release from enteric neurons. If Bethanechol does not increase post-prandial glucagon release in humans with T2DM, then neurotransmitters other than acetylcholine relay the effects of xenin-25 on glucagon release.

Screening and selection of subjects is essentially as described earlier. Studies will be conducted using subjects with NGT, IGT, and T2DM (up to 15 subjects per group which also allows for dropouts) defined by the 2-hour plasma glucose level in the screening 75-g oral glucose tolerance test. ***The use of choline esters is contraindicated in persons with hyperthyroidism, coronary artery disease, peptic ulcer, asthma, chronic bronchitis, or COPD. Subjects with any of these conditions (or other exclusions listed on pages 2-3) will be excluded from this study.*** Since this Study Protocol does NOT involve infusion of xenin-25, subjects who participated in our previous 2 studies (Protocols 1 and 2) will be allowed to enroll in this study.

Experimental Design is essentially the same as described for the Boost Plus study detailed in Protocol 2. The major difference is that instead of peptide infusions, subjects will be administered escalating doses of bethanechol, a muscarinic agonist. Each dose will be administered on a separate occasion. To monitor for symptoms of Bethanechol toxicity (i.e. parasympathetic activity, subjects will be placed on telemetry with blood pressure checked every 15 minutes. Should bradycardia <45beats per minute or hypotension (SBP<9) develop, the study will be stopped. If subjects develop nausea and vomiting or excessive diarrhea that in the opinion of the subject or PI is unacceptable, the infusion will also be stopped.

Dosing for bethanechol is based on a survey of the literature (9,10). Bethanechol is structurally related to acetylcholine, does not does not cross the blood brain barrier, and therapeutic doses act only on muscarinic receptors in the periphery. Dose-response studies have been conducted in humans using the stretch response of the bladder muscle as a readout. A progressive increase in mean cystometric pressure was noted with doses of 50, 100, and 200 mg P.O. Of the 20 subjects in this study, sweating, diarrhea, chills, bradycardia and hypotension were noted in only 1-2 patients with the 200 mg dose. Maximal response to oral bethanechol began after ~1 hour and the action of moderate doses (50-100 mg) persisted for 2-3 hours. Bethanechol has also been used without serious side effects to treat humans with xerostomia after radiation therapy [25-50 mg 3 times per day (11,12)] and to relieve side effects due to tricyclic antidepressants [25 mg 3 times per day (13)]. Based on these studies, we feel that we can safely administer single doses of bethanechol at up to 100 mg P.O. Any individual who experiences serious adverse cholinergic effects (e.g. respiratory distress, GI spasm, vomiting, severe diarrhea, cardiac arrhythmia, hypotension) will not receive any higher doses of bethanechol. If our initial studies suggest that lower doses of bethanechol may be effective, we will reduce the administered dose to 12.5mg.

Since the effects of xenin-25 on insulin release and plasma glucagon levels are greatest within the first 30-60 minutes after ingestion of a meal, the short half life of Bethanechol will not prevent us from obtaining useful data.

<u>Study Visit 1</u>: Blood for basal measurements of plasma glucose, glucagon, insulin, C-peptide, GIP, and GLP-1 will be collected at 15 minute intervals beginning at minus 90 minutes. At minus 60 minutes, subjects will be administered a placebo P.O. At time zero, subjects will ingest Boost Plus and acetaminophen after which, blood will be sampled for measurements of plasma glucose, peptides and acetaminophen as described in Protocol 2. Since plasma amylase and lipase levels have never been elevated as a results of peptide infusions (60 subjects/120 infusions with xenin), plasma amylase will no longer be measured and blood for CMP will be taken only at the -90 minute time point. This will require 226 mL of blood per visit. Visits will be scheduled so that less than 550 mL blood is collected in any 8 week period.

<u>Study Visit 2</u>: This visit will be the same as visit 1 except one hour before ingestion of the meal, subjects will be administered 25 mg Bethanechol P.O. instead of placebo.

<u>Study Visit 3</u>: This visit will be the same as visit 1 except one hour before ingestion of the meal, subjects will be administered 50 mg Bethanechol P.O. instead of placebo.

<u>Study Visit 4</u>: This visit will be the same as visit 1 except one hour before ingestion of the meal, subjects will be administered 100 mg Bethanechol P.O. instead of placebo.

Approach to Data Analysis and Anticipated Results.

Data analysis is essentially as described for Protocol 2. Based on pre-clinical studies as well as our interim results using Protocol 2, it is predicted that bethanechol will increase insulin secretion rates in all subjects but profoundly increase plasma glucagon levels only in humans with T2DM. If bethanechol does not increase these outcomes, then the effects of enteric neurons on insulin secretion and glucagon release are different in mice and humans. It is also possible that bethanechol will increase only insulin or glucagon release, but not both.

Reference List

1. Kissebah,AH, Vydelingum,N, Murray,R, Evans,DJ, Hartz,AJ, Kalkhoff,RK, Adams,PW: Relation of body fat distribution to metabolic complications of obesity. *J Clin Endocrinol Metab* 54:254-260, 1982
2. Bjorntorp,P: Obesity and risk of cardiovascular disease. *Acta Med Scand* 218:145-147, 1985
3. Despres,JP, Moorjani,S, Lupien,PJ, Tremblay,A, Nadeau,A, Bouchard,C: Regional distribution of body fat, plasma lipoproteins, and cardiovascular disease. *Arteriosclerosis* 10:497-511, 1990
4. Report of the Expert Committee on the Diagnosis and Classification of Diabetes Mellitus. *Diabetes Care* 20:1183-1197, 1997
5. Feurle,GE, Hamscher,G, Kusiek,R, Meyer,HE, Metzger,JW: Identification of xenin, a xenopsin-related peptide, in the human gastric mucosa and its effect on exocrine pancreatic secretion. *J Biol Chem* 267:22305-22309, 1992
6. Toffolo,G, De,GF, Cobelli,C: Estimation of beta-cell sensitivity from intravenous glucose tolerance test C-peptide data. Knowledge of the kinetics avoids errors in modeling the secretion. *Diabetes* 44:845-854, 1995
7. Breda,E, Cavaghan,MK, Toffolo,G, Polonsky,KS, Cobelli,C: Oral glucose tolerance test minimal model indexes of beta-cell function and insulin sensitivity. *Diabetes* 50:150-158, 2001
8. Barrett,PH, Bell,BM, Cobelli,C, Golde,H, Schumitzky,A, Vicini,P, Foster,DM: SAAM II: Simulation, Analysis, and Modeling Software for tracer and pharmacokinetic studies. *Metabolism* 47:484-492, 1998
9. Diokno,AC, Lapides,J: Action of oral and parenteral bethanechol on decompensated bladder. *Urology* 10:23-24, 1977
10. Lapides,J, FRIEND,CR, JEMIAN,EP, SONDA,LP: COMPARISON OF ACTION OF ORAL AND PARENTERAL BETHANECHOL CHLORIDE UPON THE URINARY BLADDER. *Invest Urol* 1:94-97, 1963
11. Gorsky,M, Epstein,JB, Parry,J, Epstein,MS, Le,ND, Silverman S Jr: The efficacy of pilocarpine and bethanechol upon saliva production in cancer patients with hyposalivation following radiation therapy. *Oral Surg Oral Med Oral Pathol Oral Radiol Endod* 97:190-195, 2004
12. Jham,BC, Teixeira,IV, Aboud,CG, Carvalho,AL, Coelho,MM, Freire,AR: A randomized phase III prospective trial of bethanechol to prevent radiotherapy-induced salivary gland damage in patients with head and neck cancer. *Oral Oncol* 43:137-142, 2007
13. Everett,HC: The use of bethanechol chloride with tricyclic antidepressants. *Am J Psychiatry* 132:1202-1204, 1975
